# Synthesis of Some Oxadiazole Derivatives as New Anticandidal Agents

**DOI:** 10.3390/molecules16097662

**Published:** 2011-09-07

**Authors:** Zafer Asim Kaplancikli

**Keywords:** oxadiazole, pyrimidine, anticandidal activity

## Abstract

In this study, 5-[(pyrimidin-2-ylthio)methyl]-1,3,4-oxadiazole-2(3H)-thione (**3**) was synthesized via the ring closure reaction of 2-(pyrimidin-2-ylthio)acetohydrazide (**2**) with carbon disulphide. New oxadiazole derivatives **4a–f** were obtained by the nucleophilic substitution reaction of compound **3** with various phenacyl bromides. The chemical structures of the compounds were elucidated by IR, ^1^H-NMR, ^13^C-NMR and FAB^+^-MS spectral data and elemental analyses. The newly synthesized derivatives **4a–f** were tested *in vitro* by using a microbroth dilution method against *C. albicans* (clinical isolate, Osmangazi University, Faculty of Medicine, Eskişehir, Turkey), *C. albicans* (ATCC 90028), *C. glabrata* (clinical isolate, Osmangazi University, Faculty of Medicine, Eskişehir, Turkey), *C. tropicalis* (NRRL Y-12968), *C. krusei* (NRRL Y-7179), *C. parapsilosis* (NRRL Y- 12696), *C. albicans* (NRRL Y-12983), *C. glabrata* (clinical isolate, Anadolu University, Faculty of Science, Department of Biology, Eskişehir, Turkey). Among these compounds, compound **4a** was found to be the most potent derivative (MIC = 0.007–0.06 versus ketoconazole: 0.001–0.007 mg/mL) against *Candida* species, except *C. tropicalis* and *C. krusei* when compared with the standard antifungal ketoconazole.

## 1. Introduction

The incidence of invasive fungal infections, particularly those related to *Candida* species, has increased worldwide over the past two decades. It is obvious that *Candida* has emerged as one of the leading causes of nosocomial bloodstream infections. Although there are some antifungal agents currently available, the treatment of invasive fungal infections still remains a challenging problem due to the development of resistance accompanying the widespread use of these drugs. As a consequence of this situation, medicinal chemists have focused on the development of more effective agents for the treatment of invasive fungal infections caused by resistant organisms [1,2,3,4,5].

Oxadiazoles have gained great importance in medicinal chemistry owing to their broad spectrum and metabolic profile [6]. Many studies have confirmed that oxadiazole derivatives possess antifungal activity [7,8,9,10,11,12,13,14]. Among oxadiazole derivatives, 1,3,4-oxadiazolin-2-thiones have received a great deal of attention in heterocyclic chemistry as versatile intermediates due to the fact that the thiol group on oxadiazole ring undergoes nucleophilic substitution reactions readily [15,16].

Pyrimidine derivatives have been found to possess anticandidal activity. Voriconazole, which is an azole derivative bearing pyrimidine group, is widely used as an antifungal agent for the treatment of candidiasis [5,17,18,19]. On the other hand, some researchers have also carried out considerable research for novel antifungal agents bearing thioether moiety. The prominent compounds bearing thioether group are butoconazole and sulconazole, all of which are widely used as antifungal drugs for the treatment of candidiasis [20,21,22].

In the present study, new oxadiazole derivatives bearing two important functional moieties, namely pyrimidine and thioether, were synthesized and furthermore, their anticandidal effects were elaborated. 

## 2. Results and Discussion

**Scheme 1:**
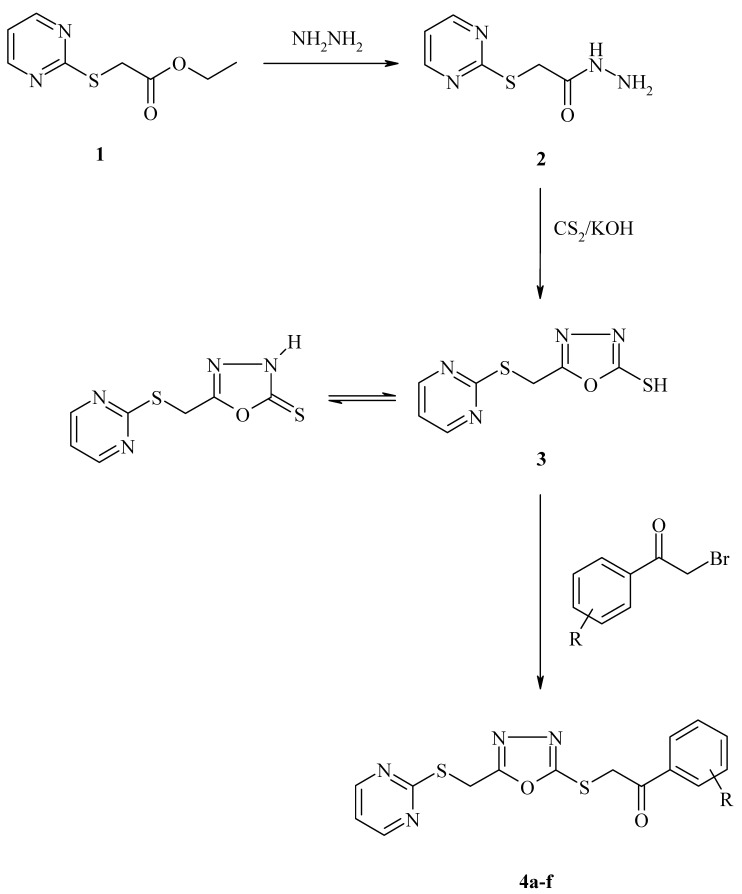
The synthetic protocol of the compounds (**4a–f**).

Initially, 5-[(pyrimidin-2-ylthio)methyl]-1,3,4-oxadiazole-2(3H)-thione (**3**) was obtained by the ring closure reaction of the acid hydrazide **2** with carbon disulphide in the presence of potassium hydroxide. The target compounds **4a–f** were synthesized via the nucleophilic substitution reaction of 5-[(pyrimidin-2-ylthio)methyl]-1,3,4-oxadiazole-2(3H)-thione (**3**) with various phenacyl bromides. These reactions are summarized in Scheme 1 and some properties of the compounds are given in Table 1.

The structures of the compounds **4a–f** were confirmed by IR, ^1^H-NMR, ^13^C-NMR and FAB-MS spectral data and Elemental analyses. In the IR spectra of all compounds **4a–f**, all derivatives have a strong, characteristic band in the region 1685–1675 cm^−1^ due to the C=O stretching vibration. The bands due to C=C and C=N stretching vibrations are observed in the region 1620–1400 cm^−1^.

**Table 1:** Some properties of the compounds (**4a–f**).

**Compound**	R	Yield (%)	M.p. (°C)	Molecular formula	Molecular weight
**4a**	H	75	112-114	C_15_H_12_N_4_O_2_S_2_	344
**4b**	p-Cl	85	106-108	C_15_H_11_ClN_4_O_2_S_2_	378,5
**4c**	p-NO_2_	95	145-149	C_15_H_11_N_5_O_4_S_2_	389
**4d**	m-NO_2_	92	79-80	C_15_H_11_N_5_O_4_S_2_	389
**4e**	m-Cl	84	89-90	C_15_H_11_ClN_4_O_2_S_2_	378,5
**4f**	2,4-Cl	88	75-77	C_15_H_10_Cl_2_N_4_O_2_S_2_	413

In the ^1^H NMR spectra of all compounds **4a–f**, the signals due to the -S-CH_2_- protons directly attached to oxadiazole ring and carbonyl group appeared at 4.67–4.72 ppm and 4.91–5.17 ppm, respectively. The pyrimidine ring protons were observed in the region 7.25–8.72 ppm. The other aromatic protons were observed at expected regions. 

In their ^13^C-NMR spectra of **4a–f**, the signals due to the carbonyl carbons were observed at 191–193 ppm. The oxadiazole ring carbons appeared at 163.61–165.77 ppm. The aromatic carbons were observed in the expected regions. 

In the mass spectra of all compounds **4a–f**, the M+1 peak was observed. All compounds gave satisfactory elemental analysis.

All compounds were tested *in vitro* against *C. albicans* (clinical isolate, Osmangazi University, Faculty of Medicine, Eskişehir, Turkey), *C. albicans* (ATCC 90028), *C. glabrata* (clinical isolate, Osmangazi University, Faculty of Medicine, Eskişehir, Turkey), *C. tropicalis* (NRRL Y-12968), *C. krusei* (NRRL Y-7179), *C. parapsilosis* (NRRL Y- 12696), *C. albicans* (NRRL Y-12983), *C. glabrata* (clinical isolate, Anadolu University, Faculty of Science, Department of Biology, Eskişehir, Turkey) and compared with ketoconazole (Table 2). 

**Table 2:** Anticandidal activities of novel compounds (**4a–f**) as MIC values (mg/mL).

	4a	4b	4c	4d	4e	4f	Ref.
**A**	0.015	0.015	0.06	0.125	0.015	0.03	0.007
**B**	0.03	0.03	0.03	0.06	0.03	0.06	0.007
**C**	0.03	0.125	0.06	0.06	0.125	0.06	0.003
**D**	0.015	0.015	0.015	0.06	0.06	0.06	0.001
**E**	0.007	0.03	0.015	0.125	0.03	0.015	0.001
**F**	0.06	0.06	0.03	0.06	0.06	0.06	0.001
**G**	0.06	0.06	0.06	0.125	0.06	0.06	0.001
**H**	0.06	0.03	0.03	0.06	0.03	0.015	0.001

**A**: *C. albicans* (clinical isolate, Osmangazi University, Faculty of Medicine, Eskişehir, Turkey), **B**: *C. albicans* (ATCC 90028), **C**: *C. albicans* (NRRL Y-12983), **D**: *C. glabrata* (clinical isolate, Osmangazi University, Faculty of Medicine, Eskişehir, Turkey), **E**: *C. glabrata* (clinical isolate, Anadolu University, Faculty of Science, Department of Biology, Eskişehir, Turkey), **F**: *C. krusei* (NRRL Y-7179), **G**: *C. parapsilosis* (NRRL Y- 12696), **H**: *C. tropicalis* (NRRL Y-12968), **Ref.**:Ketoconazole.

The biological results indicate that *C. albicans* (clinical isolate) is the most susceptible fungus to compounds **4a**, **4b** and **4e**. These compounds exhibit the strongest inhibitory activity against *C. albicans* (clinical isolate) with a MIC value of 0.015 mg/mL, when compared with the standard antifungal ketoconazole, which exhibits inhibitory activity with a MIC value of 0.007 mg/mL. This outcome confirms that phenyl and chlorophenyl groups may have a considerable influence on antifungal activity against the tested clinical *C. albicans* isolate. It can be attributed to + π effect of phenyl and chlorophenyl groups. Compounds **4c** and **4d** exhibit lower inhibitory activity against *C. albicans* (clinical isolate) which may be due to—π effect of the nitro substituent.

The standard strain *C. albicans* (ATCC 90028) showed an inhibition range with MIC values of 0.03–0.06 mg/mL of the tested compounds **4a–f** suggesting moderate inhibitory activity compared to the clinical isolate versus ketoconazole. The other tested *C. albicans* strain (NRRL Y-12983) was less susceptible to the tested compounds, with the MIC = 0.03–0.125 mg/mL. 

The tested compounds **4a–c** showed strong inhibitory activity (0.03 mg/mL) against and *C. glabrata* (clinical isolate, Eskisehir Osmangazi University, Eskişehir, Turkey), and relatively good inhibition was also observed in compounds **4d–f** with an inhibition value of 0.06 mg/mL. However, compound **4a** exhibits the most potent inhibitory activity among the tested substances against *C. glabrata* (clinical isolate, Anadolu University, Faculty of Science, Department of Biology, Eskişehir, Turkey) with a MIC value of 0.007 mg/mL, whereas ketoconazole exhibits inhibitory activity with a MIC value of 0.001 mg/mL. The *Candida* species was susceptible to compounds **4b–f** with MIC values of 0.015–0.125 mg/mL.

Compound **4c** is the most potent (MIC = 0.03 mg/mL) derivative against *C. krusei* (NRRL Y-7179). It is obvious that *p*-nitro group has an important impact on antifungal activity against *C. krusei*. Other tested compounds showed an inhibition effect with MIC 0.06 mg/mL. 

The standard strain *C. parapsilosis* (NRRL Y- 12696) was inhibited by all tested compounds relatively well, with an MIC value of 0.06 mg/mL, except compound **4d** with an MIC value of 0.125 mg/mL, whereas ketoconazole showed an inhibition concentration of 0.001 mg/mL.

Compound **4f** exhibits the highest antifungal activity (MIC 0.015 mg/mL) against * C. tropicalis* (NRRL Y-12968) compared to ketoconazole (MIC 0.001 mg/mL) among the tested compounds against this pathogen. It is apparent that there is a positive correlation between antifungal activity against *C. tropicalis* and 2,4-dichlorophenyl substituent.

## 3. Experimental

### 3.1. General

All reagents were used as purchased from commercial suppliers without further purification. Melting points were determined on an Electrothermal 9100 digital melting point apparatus and were uncorrected (Electrothermal, Essex, UK). Spectroscopic data were recorded on the following instruments: IR, Shimadzu 435 IR spectrophotometer (Shimadzu, Tokyo, Japan) ^1^H-NMR (400 MHz) and ^13^C-NMR (100 MHz) Bruker (Avance DRX-400) NMR spectrometer (Bruker Bioscience, Billerica, MA, USA) in DMSO-d_6_ using TMS as internal standard; MS-FAB, VG Quattro mass spectrometer (Fisons Instruments Vertriebs GmbH, Mainz, Germany). Elemental analyses were performed on a Perkin Elmer EAL 240 elemental analyser (Perkin-Elmer, Norwalk, CT, USA).

### 3.2. General Procedure for Synthesis of the Compounds

*Ethyl 2-(pyrimidin-2-ylthio)acetate* (**1**). A mixture of 2-mercaptopyrimidine (0.05 mol) and ethyl chloroacetate (0.05 mol) in the presence of potassium carbonate (0.05 mol) in acetone (100 mL) was refluxed for 10 h. The reaction mixture was cooled, filtered and the crude product was solved in water and then extracted with ether [17].

*2-(Pyrimidin-2-ylthio)acetohydrazide* (**2**). A mixture of the ester (**1**, 0.05 mol) and hydrazine hydrate (0.1 mol) in ethanol (100 mL) was stirred at room temperature for 3 hours and then filtered [17].

*5-[(pyrimidin-2-ylthio)methyl]-1,3,4-oxadiazole-2(3H)-thione* (**3**). A mixture of the hydrazide (**2**) (0.01 mol) and carbon disulfide (10 mL) in the presence of potassium hydroxide (0.01 mol) in ethanol (15 mL) was refluxed for 10 h. The solution was cooled and acidified to pH 5-6 with hydrochloric acid solution and crystallized from ethanol [17].

*2-[5-[(Pyrimidin-2-ylthio)methyl] -1,3,4-oxadiazol-2-ylthio]acetophenone derivatives* **4a–f**. 5-[(Pyrimidin-2-ylthio)methyl]-1,3,4-oxadiazole-2(3H)-thione (**3**) (0.01 mol) and appropriate phenacyl bromide (0.01 mol) in acetone (15 mL) were stirred at room temperature for 8 hours and filtered. The residue was crystallized from ethanol.

*2-[5-[(Pyrimidin-2-ylthio)methyl] -1,3,4-oxadiazol-2-ylthio]acetophenone* (**4a**). IR (KBr) ν_max_ (cm^−1^): 3057 (aromatic C-H), 1683 (C=O), 1605, 1551, 1440 (C=N and C=C). ^1^H-NMR: 4.68 (2H, s, CH_2_-oxadiazole), 5.08 (2H, s, CH_2_-CO), 7.25 (1H, dd, pyrimidine), 7.56–7.60 (2H, dd, phenyl), 7.69 (1H, dd, phenyl), 8.01–8.03 (2H, d, phenyl), 8.65–8.66 (2H, d, pyrimidine); ^13^C-NMR: 31.17 (CH_2_, CH_2_-oxadiazole), 40.93 (CH_2_, CH_2_-CO), 118.41 (CH, pyrimidine), 128.89 (2CH, phenyl), 129.36 (2CH, phenyl), 134.44 (CH, phenyl), 135.45 (C, phenyl), 158.51 (CH, pyrimidine), 158.61 (CH, pyrimidine), 163.88 (C, oxadiazole), 165.64 (C, oxadiazole), 169.11 (C, pyrimidine), 192.75 (C, C=O); Anal. For C_15_H_12_N_4_O_2_S_2_ calculated: C, 52.31; H, 3.51; N, 16.27; found: C, 52.30; H, 3.52; N, 16.25; MS (FAB) [M+1]^+^: m/z 345.

*2-[5-[(Pyrimidin-2-ylthio)methyl] -1,3,4-oxadiazol-2-ylthio]-4′-chloroacetophenone* (**4b**). IR (KBr) ν_max_ (cm^−1^): 3066 (aromatic C-H), 1685 (C=O), 1615, 1560, 1445 (C=N and C=C). ^1^H-NMR: 4.67 (2H, s, CH_2_-oxadiazole), 5.06 (2H, s, CH_2_-CO), 7.25 (1H, dd, pyrimidine), 7.61–7.67 (2H, d, 4-chlorophenyl), 7.99–8.05 (2H, d, 4-chlorophenyl), 8.65–8.71 (2H, d, pyrimidine); ^13^C-NMR: 31.10 (CH_2_, CH_2_-oxadiazole), 40.80 (CH_2_, CH_2_-CO), 118.40 (CH, pyrimidine), 129.48 (2CH, 4-chlorophenyl), 130.81 (2CH, 4-chlorophenyl), 134.15 (C, 4-chlorophenyl), 139.39 (C, 4-chlorophenyl), 158.51 (2CH, pyrimidine), 163.88 (C, oxadiazole), 165.68 (C, oxadiazole), 169.90 (C, pyrimidine), 191.93 (C, C=O); Anal. For C_15_H_11_ClN_4_O_2_S_2_ calculated: C, 47.55; H, 2.93; N, 14.79; found: C, 47.52; H, 2.90; N, 14.81; MS (FAB) [M+1]^+^: m/z 379.

*2-[5-[(Pyrimidin-2-ylthio)methyl] -1,3,4-oxadiazol-2-ylthio]-4′-nitroacetophenone* (**4c**). IR (KBr) ν_max_ (cm^−1^): 3067 (aromatic C-H), 1685 (C=O), 1604, 1554, 1442 (C=N and C=C). ^1^H-NMR: 4.72 (2H, s, CH_2_-oxadiazole), 5.13 (2H, s, CH_2_-CO), 7.25 (1H, dd, pyrimidine), 8.15–8.26 (2H, d, 4-nitrophenyl), 8.28–8.42 (2H, d, 4-nitrophenyl), 8.60–8.71 (2H, d, pyrimidine); ^13^C-NMR: 33.51 (CH_2_, CH_2_-oxadiazole), 41.05 (CH_2_, CH_2_-CO), 118.41 (CH, pyrimidine), 124.41 (2CH, 4-nitrophenyl), 130.34 (2CH, 4-nitrophenyl), 140.10 (C, 4-nitrophenyl), 150.75 (C, 4-nitrophenyl), 158.52 (CH, pyrimidine), 158.61 (CH, pyrimidine), 163.61 (C, oxadiazole), 165.77 (C, oxadiazole), 169.09 (C, pyrimidine), 192.29 (C, C=O); Anal. For C_15_H_11_N_5_O_4_S_2_ calculated: C, 46.27; H, 2.85; N, 17.98; found: C, 46.30; H, 2.82; N, 18.00; MS (FAB) [M+1]^+^: m/z 390.

*2-[5-[(Pyrimidin-2-ylthio)methyl] -1,3,4-oxadiazol-2-ylthio]-3′-nitroacetophenone* (**4d**). IR (KBr) ν_max_ (cm^−1^): 3067 (aromatic C-H), 1684 (C=O), 1605, 1555, 1440 (C=N and C=C). ^1^H-NMR: 4.67 (2H, s, CH_2_-oxadiazole), 5.17 (2H, s, CH_2_-CO), 7.26 (1H, dd, pyrimidine), 7.87–7.91 (1H, dd, 3-nitrophenyl), 8.43–8.54 (2H, d, 3-nitrophenyl), 8.59–8.61 (1H, s, 3-nitrophenyl), 8.63–8.72 (2H, d, pyrimidine); ^13^C-NMR: 31.15 (CH_2_, CH_2_-oxadiazole), 40.82 (CH_2_, CH_2_-CO), 118.39 (CH, pyrimidine), 123.26 (CH, 3-nitrophenyl), 128.55 (CH, 3-nitrophenyl), 131.22 (CH, 3-nitrophenyl), 135.06 (CH, 3-nitrophenyl), 136.66 (C, 3-nitrophenyl), 148.52 (C, 3-nitrophenyl), 158.51 (CH, pyrimidine), 158.61 (CH, pyrimidine), 163.61 (C, oxadiazole), 165.77 (C, oxadiazole), 169.10 (C, pyrimidine), 191.72 (C, C=O); Anal. For C_15_H_11_N_5_O_4_S_2_ calculated: C, 46.27; H, 2.85; N, 17.98; found: C, 46.28; H, 2.86; N, 17.99; MS (FAB) [M+1]^+^: m/z 390.

*2-[5-[(Pyrimidin-2-ylthio)methyl] -1,3,4-oxadiazol-2-ylthio]-3′-chloroacetophenone* (**4e**). IR (KBr) ν_max_ (cm^−1^): 3065 (aromatic C-H), 1685 (C=O), 1614, 1555, 1445 (C=N and C=C). ^1^H-NMR: 4.67 (2H, s, CH_2_-oxadiazole), 5.07 (2H, s, CH_2_-CO), 7.25 (1H, dd, pyrimidine), 7.59-7.66 (1H, dd, 3-chlorophenyl), 7.68-7.79 (1H, d, 3-chlorophenyl), 7.84-7.85 (1H, d, 3-chlorophenyl), 7.92-8.04 (1H, s, 3-chlorophenyl), 8.60-8.71 (2H, d, pyrimidine); ^13^C-NMR: 31.20 (CH_2_, CH_2_-oxadiazole), 40.77 (CH_2_, CH_2_-CO), 118.39 (CH, pyrimidine), 127.53 (CH, 3-chlorophenyl), 128.58 (CH, 3-chlorophenyl), 129.21 (CH, 3-chlorophenyl), 131.35 (CH, 3-chlorophenyl), 134.10 (C, 3-chlorophenyl), 137.27 (C, 3-chlorophenyl), 158.50 (CH, pyrimidine), 158.61 (CH, pyrimidine), 163.70 (C, oxadiazole), 165.71 (C, oxadiazole), 169.00 (C, pyrimidine), 191.93 (C, C=O); Anal. For C_15_H_11_ClN_4_O_2_S_2_ calculated: C, 47.55; H, 2.93; N, 14.79; found: C, 47.52; H, 2.90; N, 14.80; MS (FAB) [M+1]^+^: m/z 379.

*2-[5-[(Pyrimidin-2-ylthio)methyl] -1,3,4-oxadiazol-2-ylthio]-2′,4′-*dichloroacetophenone (**4f**). IR (KBr) ν_max_ (cm^−1^): 3065 (aromatic C-H), 1685 (C=O), 1620, 1552, 1415 (C=N and C=C). ^1^H-NMR: 4.68 (2H, s, CH_2_-oxadiazole), 4.91 (2H, s, CH_2_-CO), 7.27 (1H, dd, pyrimidine), 7.53–7.64 (2H, 2,4-dichlorophenyl), 7.73–7.88 (1H, d, 2,4-dichlorophenyl), 8.61–8.70 (2H, d, pyrimidine); ^13^C-NMR: 33.58 (CH_2_, CH_2_-oxadiazole), 42.71 (CH_2_, CH_2_-CO), 118.53 (CH, pyrimidine), 128.26 (CH, 2,4-dichlorophenyl), 128.55 (CH, 2,4-dichlorophenyl), 130.93 (C, 2,4-dichlorophenyl), 131.51 (CH, 2,4-dichlorophenyl), 135.23 (C, 2,4-dichlorophenyl), 137.59 (C, 2,4-dichlorophenyl), 158.54 (CH, pyrimidine), 158.61 (CH, pyrimidine), 163.47 (C, oxadiazole), 165.77 (C, oxadiazole), 169.11 (C, pyrimidine), 193.73 (C, C=O); Anal. For C_15_H_10_Cl_2_N_4_O_2_S_2_ calculated: C, 43.59; H, 2.44; N, 13.56; found: C, 43.60; H, 2.45; N, 13.53; MS (FAB) [M+1]^+^: m/z 414.

### 3.3. Microbiology

#### 3.3.1. Anticandidal Evaluation

A modified microbroth dilution method was carried out according to the procedure [23,24]. The compounds **4a–f** were tested *in vitro* against *C. albicans* (clinical isolate, Osmangazi University, Faculty of Medicine, Eskişehir, Turkey), *C. albicans* (ATCC 90028), *C. albicans* (NRRL Y-12983), *C. glabrata* (clinical isolate, Osmangazi University, Faculty of Medicine, Eskişehir, Turkey),*C. glabrata* (clinical isolate, Anadolu University, Faculty of Science, Department of Biology, Eskişehir, Turkey), *C. krusei* (NRRL Y-7179), *C. parapsilosis* (NRRL Y- 12696), *C. tropicalis* (NRRL Y-12968).

Microorganisms were stored in 15% glycerol containing micro-test tubes at −86 °C. All *Candida* strains were inoculated on Sabouraud Dextrose Agar (SDA) prior to the experiments at 37 °C. After sufficient growth, *Candida* spp. were then transferred to Mueller Hinton Broth (MHB) for further incubation under the same conditions for another 24 h.

#### 3.3.2. Microbroth Dilution Assay

The test compounds and the antifungal standard were first dissolved in dimethyl sulfoxide (DMSO) which was used to prepare the stock solutions at an initial concentration of 2 mg/mL. Serial dilution series were prepared in MHB (10 mL) with an equal amount of the test samples. The last row was filled only with water as growth control for the microorganism. Overnight grown microorganism suspensions were first diluted in double strength MHB and standardized to 1 × 10^8^ CFU/mL (using McFarland No: 0.5) under sterile conditions. Then each microorganism suspension was pippetted into each well and incubated at 37 °C for 24 h. Ketoconazole was used as a standard antifungal agent against *Candida* spp. Sterile distilled water and medium served as a positive growth control. The first well without turbidity was assigned as the minimum inhibitory concentration (MIC, in mg/mL). 

## 4. Conclusions

In conclusion, the syntheses of new oxadiazole derivatives were described, which were also tested *in vitro* against various clinical and standard *Candida* species for their antifungal effects. 5-[(Pyrimidin-2-ylthio)methyl]-1,3,4-oxadiazole-2(3H)-thione (**3**) was obtained via the ring closure reaction of the acid hydrazide **2** with carbon disulphide. New oxadiazole derivatives were prepared by the nucleophilic substitution reaction of compound **3** with appropriate phenacyl bromides. Among these compounds, the non-substituted compound **4a** is the most potent derivative (MIC: 0.007–0.06 mg/mL) against *Candida* species except *C. tropicalis* and *C. krusei*. 

Within the tested yeasts, *C. albicans* (clinical isolate) seems to be the most susceptible strain to compounds **4a**, **4b** and **4e**. These compounds exhibit inhibitory activity with a MIC value of 0.015 mg/mL, whereas ketoconazole exhibits inhibitory activity with a MIC value of 0.007 mg/mL. This could result from increased lipophilicity associated with phenyl and chlorophenyl groups. Compound **4f** exhibits the highest antifungal activity against *C. tropicalis* (NRRL Y-12968). It is clear that the 2,4-dichlorophenyl substituent has an important impact on antifungal activity against *C. tropicalis*. Compound **4c** is the most effective derivative against *C. krusei* (NRRL Y-7179). It is apparent that the *p*-nitro group may have a considerable influence on antifungal activity against this species.
